# Automated system for classifying uni-bicompartmental knee osteoarthritis by using redefined residual learning with convolutional neural network

**DOI:** 10.1016/j.heliyon.2024.e31017

**Published:** 2024-05-14

**Authors:** Soaad M. Naguib, Mohamed A. Kassem, Hanaa M. Hamza, Mostafa M. Fouda, Mohammed K. Saleh, Khalid M. Hosny

**Affiliations:** a Department of Information Systems, Faculty of Computers and Informatics, Zagazig University, Zagazig, 44519, Egypt; b Department of Robotics and Intelligent Machines, Faculty of Artificial Intelligence, Kafrelsheikh University, Kafr el-Sheikh, Egypt; c Department of Information Technology, Faculty of Computers and Informatics, Zagazig University, Zagazig, 44519, Egypt; d Department of Electrical and Computer Engineering, Idaho State University, Pocatello, ID, 83209, USA; e Department of Orthopedic Surgery, Faculty of Medicine, Zagazig University, Zagazig, 44519, Egypt

**Keywords:** Deep learning, Redefined residual learning, Knee osteoarthritis classification, Overstep connection, X-ray

## Abstract

Knee Osteoarthritis (OA) is one of the most common joint diseases that may cause physical disability associated with a significant personal and socioeconomic burden. X-ray imaging is the cheapest and most common method to detect Knee (OA). Accurate classification of knee OA can help physicians manage treatment efficiently and slow knee OA progression. This study aims to classify knee OA X-ray images according to anatomical types, such as uni or bicompartmental. The study proposes a deep learning model for classifying uni or bicompartmental knee OA based on redefined residual learning with CNN. The proposed model was trained, validated, and tested on a dataset containing 733 knee X-ray images (331 normal Knee images, 205 unicompartmental, and 197 bicompartmental knee images). The results show 61.81 % and 68.33 % for accuracy and specificity, respectively. Then, the performance of the proposed model was compared with different pre-trained CNNs. The proposed model achieved better results than all pre-trained CNNs.

## 1 Introduction

**Fig. 1:**
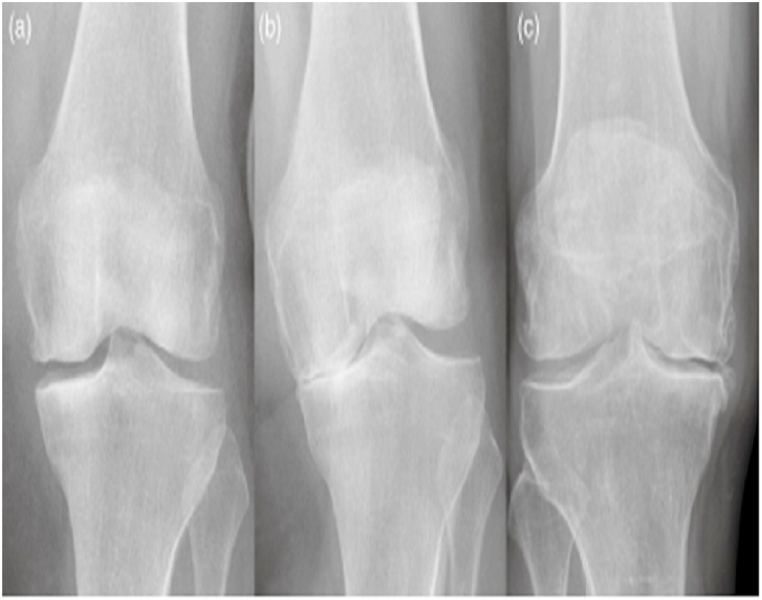
(a) Normal knee; (b) unicompartmental OA; (c) bicompartmental OA [13].

Knee OA is one of the most common joint diseases in orthopedics [1]. Knee OA is a knee joint disease accompanied by chronic pain, mobility limitations, and an increased risk of falls [2]. Knee osteoarthritis is a chronic disease with long-term symptoms and structural changes, including articular cartilage disintegration, subchondral bone sclerosis, and structural changes in all soft tissues surrounding the knee joint [[3], [4], [5]]. As a result, knee OA negatively affects the patient's quality of life due to decreased muscle strength, physical performance, range of joint motion, and physical performance [6]. Knee OA is clinically presented with limited activity, knee pain, and joint stiffness, which markedly affect the quality of life [1]. Although Knee OA mainly occurs in older adults, younger people are affected by Knee OA due to obesity and knee fractures [7]. Researchers have estimated that by 2050 around 130 million people will suffer from Knee OA [8]. The increasing need for complete knee replacements yearly reflects healthcare costs and the lack of treatment methods to prevent disease progression [9]. The Knee OA can affect two compartments of the knee, which are lateral and medial. When the OA changes only affect the medial or lateral side of the knee, this is considered a unicompartmental type. If the structural changes affect both medial and lateral sides, it is known as the bicompartmental type. Identifying uni-bicompartmental OA is valuable in determining which type of knee replacement is needed [[10], [11], [12]]. Fig. 1 (a,b, and c) represents the knee in normal, unicompartmental OA, and bicompartmental OA, respectively. X-ray imaging is one of the most common and cheap methods used in clinical medicine to scan any bone in the human body [13,14] due to its cost-effectiveness, safety, speed, and accessibility. X-rays are often used to diagnose and assess knee OA. They are the gold standard for knee OA screening [15].

The recent advances in artificial intelligence (AI) for healthcare have led to significant improvements and discoveries in orthopedics [16]. The efficiency of AI allows for quick processing of patient image data, which facilitates timely diagnosis and patient management [17]. Therefore, many researchers use AI to build computer-aided diagnosing tools to help physicians decrease time and effort while examining patients with knee OA symptoms, as many Radiographic and physicians suffer from the increasing workload in radiology and orthopedic departments [18].

[19] built a hybrid deep learning model for early diagnosing Knee OA. Three different CNN architectures were used as the base for the hybrid model. The model was compared to eight different CNN architectures, achieving the highest accuracy performance [8]. identified the grade of severity of Knee OA from X-ray images using deep learning (pre-trained CNN).

Furthermore, a conventional machine learning classifier was employed to take advantage of the enriched feature space and improve the classification performance of Knee OA. The proposed models are evaluated and prove their contribution to the early classification of the disease with a 90.8 % accuracy rate [20]. developed a deep convolutional neural network (DenseNet169) architecture coupled with an adaptive early stopping technique that uses gradual cross-entropy loss estimation for knee OA detection using X-ray images. Then, the authors compared the results obtained from the existing studies. The comparison shows that the proposed model performed better in accuracy, precision, and recall.

However, to our best knowledge, no previous studies have been done to classify Knee OA X-ray images according to anatomical types. The proposed research classifies knee OA as uni or bicompartmental. Total knee joint replacement is expensive for the patient and the national economy. Therefore, detecting that knee OA is unicompartmental will help minimize the rate of bicompartmental arthroplasty (total knee joint replacement), decreasing hospital stay and early return to daily activities. In addition, such a study can help doctors in remote areas that lack qualified orthopedic surgeons and are only supported by family and/or general practitioners.

The main contributions of this study are:

1 Classifying knee OA based on anatomical types to uni-bicompartmental types.
2 A deep learning model using an overstepping connection is developed to classify uni-bicompartmental knee OA.
3 The proposed deep learning method is based on redefined residual learning.

The remainder of the paper is organized as follows: Section 2 introduces the method used in the study. Section 3 discusses the proposed algorithm in detail. Then, we describe the used datasets by clarifying their references and explaining their classification in section 4. Then, the results and discussion of the experiment are presented in section 5. The paper is concluded in Section 6.

## 2 Methods

In knee joints, localizing information would acquire more discriminative features. It is possible to enhance classification performance by using discriminative features. When combined with the class activation map, CNNs have a substantial locating capability. CNNs have a significant locating capacity when used with the class activation map [[21], [22], [23], [24], [25]]. A class activation map was used to distinguish the discriminative ROI. Instead of relying on the pre-trained models to locate the knee, the proposed method can use different filter sizes and a cross-channel correlation approach that ignores the spatial dimensions of the ROI in a 1 × 1 convolution through the residual block to locate the ROI better. Based on the global characteristics of the same class, the proposed model annotates classification levels to focus on the explicit minimization of interclass distances. Ethical approval was waived as the data obtained was retrospective and de-identified.

### 2.1 The proposed algorithms

The classification will be incorrect if there aren't enough features employed. For a successful classification process, discriminative features are therefore crucial. A suggested deep learning approach utilizes redefined residual learning, cross-channel correlation, and an imbalanced dataset to analyze a challenging dataset of knee osteoarthritis images with uni-bicompartmental involvement. Knee-Net, a new DL architecture with 54 layers, is proposed. The recommended approach for redefined residual deep learning involves using various layers such as input, convolutional, batch normalization, pooling, dropout, fully connected, SoftMax, and activation rectified linear unit (ReLU).

The Height (H), Width (W), and Number of Channels (D) of the input image are the three dimensions that the first layer (also known as the “input layer”) is responsible for defining. In contrast to cutting-edge DL models like Google-net, Alex-net, VGG, and Res-net, limited values for W and H are employed, such as 227 × 227 or 224 × 224. All images in the W × H × D proposed model were scaled down to 300 × 300 × 3.

From the input layer, the “convolution layer” receives input. Neurons in this layer connect various image areas. Convolution layers are first learned using low-level information, after which deeper layers extract additional features, including objects, shapes, and colors. Convolutional layers are used to localize features in a scanned image. Features must be extracted from the input image after the convolutional layers, resulting in a reduced feature map to downscale an image. So, a down-sampling layer addressed this problem. Consequently, we added a max-pooling layer. The pooled features map's features are duplicated in a new group of features generated by this layer.

The input dimension of each layer is altered during the training phase by changing the layer parameters. Training the DCNN is challenging as a result. The training process is delayed by the need for slower learning rates and precise parameter settings during each iteration. Batch normalization layers were used to speed up training and reduce sensitivity. The mean and variance of the input distributions are normalized using the batch normalization layer, which also removes the negative effects of the internal shift covariance. Before the ReLU alters the DCNN, there is a normalization layer. As a result, it becomes easier to coordinate changes between layers.

It is challenging to raise and deepen layers. Even with proper initialization, the gradient vanishing problem can still arise in deeper networks. Research has established that accuracy is unaffected by increasing depth, and that degradation is unavoidable [26]. As a result, efficiency does not considerably rise as network thickness increases and may be impacted by the degradation issue. More images are needed for training deeper systems because there are numerous parameters that these networks must call to generalize. The output from one layer of a deep neural network is fed into the next layer, which is organized progressively. Going deeper is not an option because there aren't enough images in the datasets for medical images, particularly knee X-rays.

**Fig. 2:**
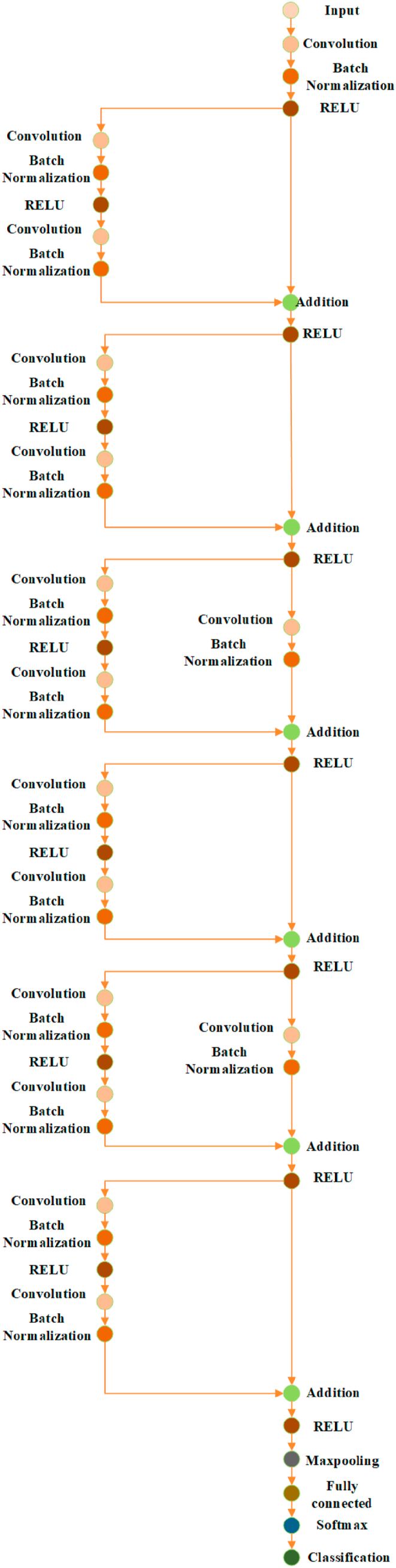
The proposed redefined residual learning deep learning model.

The proposed model employs a redefined residual learning technique to overcome image shortages and degradation. Overstepping layer input connections is a way to address degradation through redefined residual learning by increasing information flow and rearranging layers to address degradation. Different redefined residual blocks are combined to form the proposed deep redefined residual network. Fig. 2 displays the suggested method's general architecture and describes all convolutional layers.

We produce two types of redefined residual blocks, as shown in Fig. 2. Like the first, second, fourth, and sixth overstepping layer input connections in Fig. 2. , the first overstep input connections have no additional layer. The third and fifth layers of the second overstep input connections are batch normalization and convolutional. The input vector “o" is used to represent each redefined residual block, the output vector is “m,” and the mapping of the stacked layers is represented by “S(o)" for each redefined residual block. The redefined residual function of these layers is now identified using equation (1) below [22]:

(1) Ι(o)=S(o)–o

Instead of imitating the stacked layers S(o), in equation (1), the redefined residual learning is used to increase the layers' learning rate even with a small dataset. This subnetwork's output “m" is computed from equation (2):

(2) $$S\left(o_{i}\right)=Ι\left(o_{i}\right)+o_{i}$$

The proposed method has been modified to operate with multiclass classification after the fully connected and SoftMax layers. Using an entropy function, the final layer, called the output layer, changes the SoftMax output into the target class name. A probability value of 1 must be used in sigmoid. Still, a probability value 1 can be used in SoftMax if the target class has a more excellent probability value than other SoftMax classes. One potential limitation of SoftMax is that it can become computationally expensive when the number of “knee” classes increases [22].

It is also possible for the proposed method to perform a solution to the class imbalance. We developed a bootstrap algorithm to ensure the classes were balanced in the dataset. Regular sampling of images is carried out by replacing the samples with new ones, and then the samples are weighted based on how many images are in each class. The dataset images are first arranged alphabetically by the names of the classes. Now, the algorithm computes how many images are in the entire dataset and how many there are in each class. The algorithm calculated the weight of the images by dividing the count of each class image by the overall number of images in the dataset.

Furthermore, it is important to note that images within the same class are weighted equally. When all labels' weights are added together, the sum of all labels' weights equals one. The dataset is transformed into a vector, and instead of utilizing images labeled alphabetically, the labels of the images will be numerical weights. As a result, the class with the most images has the least weight, while the class with the fewest images has the highest weight. Then, the suggested technique multiplies the weight of the classification layer by the weight of the images in the image vector. The proposed redefined residual learning is shown in the following algorithm.

**Figure:**
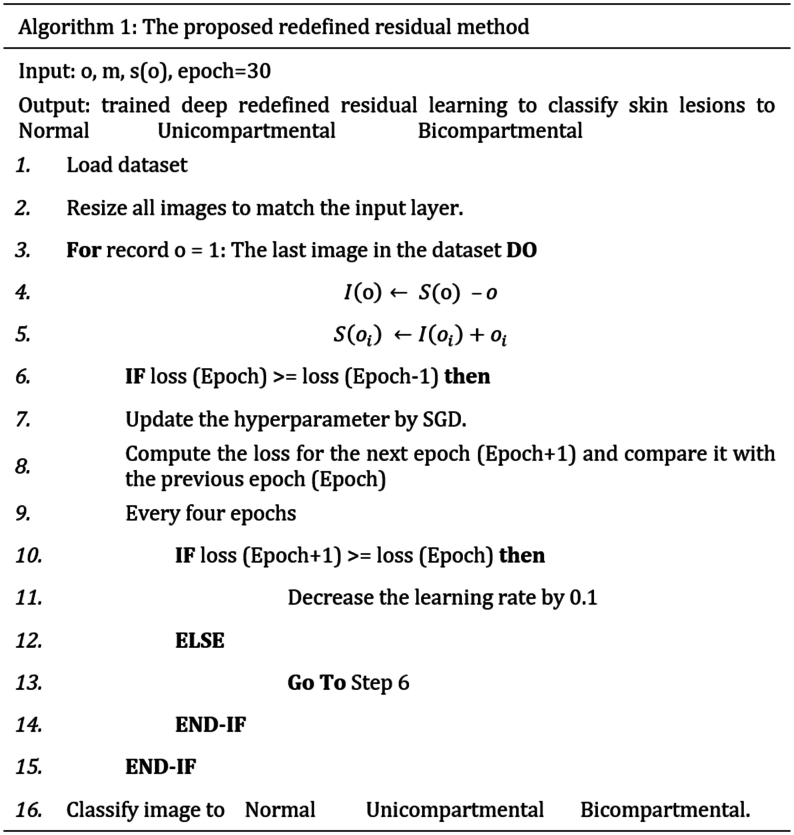

## 3 Experiment

This section will describe the used datasets by clarifying their references and explaining their classification. Then, the results of the experiments will be presented later in the following sections. The first dataset has been obtained from Mendeley Data [27]. The second dataset has been obtained from Kaggle Datasets [28]. The two image datasets have been merged into one dataset and classified into three groups. Specialized orthopedic surgeons did the classification.

**Fig. 3:**
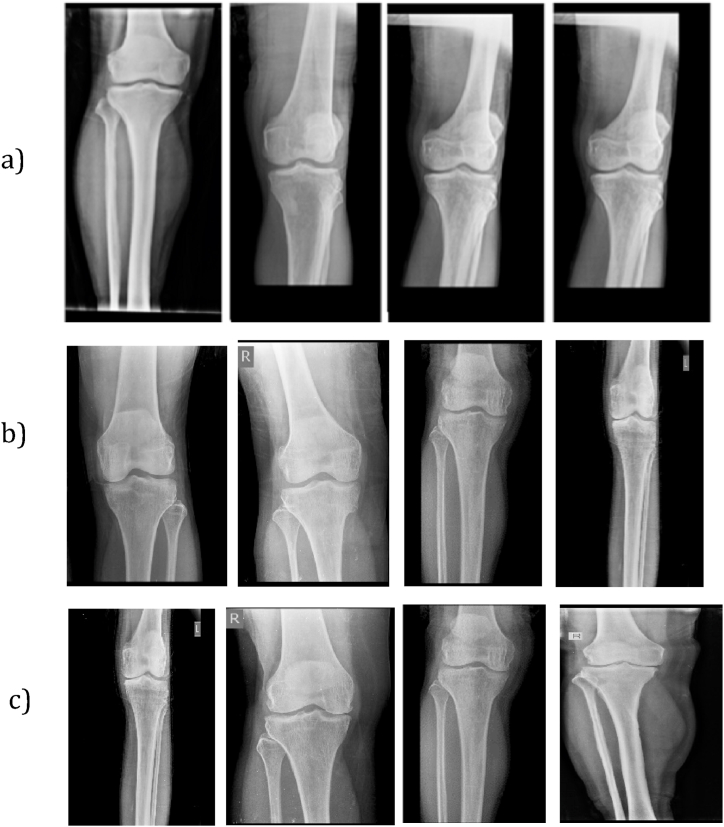
a) Samples from the normal group, b) Samples from the unicompartmental group, c) Samples from the bicompartmental group.

The first normal group includes 331 images (Fig. 3 a). The second unicompartmental group contains 205 images (Fig. 3 b). Finally, the third bicompartmental group comprises 197 images (Fig. 3 c). The proposed model was trained, validated, and tested on the merged datasets.

### 3.1 Results

The proposed model was developed in MATLAB 2022b 64-bit and executed on an IBM PC with a Core i7 CPU, 16 GB of DDRAM, and an NVIDIA MX150 GPU card. The SGD optimizer was used to adjust network weights throughout the training period. As the learning process progresses, each network weight or parameter has a specific learning rate that changes. In the training phase, the network parameters are randomly initialized and adjusted. All trials use a similar learn rate drop period, weight decay, batch size, momentum, and maximum epochs, and these values were 4, 0.9, 0.001, 10, 0.9, and 30, respectively.

Quantitative and qualitative indicators were used to evaluate the suggested model's performance. Two quantitative metrics were used: accuracy and specificity. If accuracy is used to assess the model's quality, a model that classifies all testing samples into the class with the most images will have excellent accuracy. Nonetheless, this model will not give us any helpful information. As a result, we employed other performance metrics. The confusion matrix is utilized as a qualitative metric to display and analyze the dependability of the suggested method. These measurements are calculated using the following equation [29]:

(3) $$Accuracy=\frac{t_{p}+t_{n}}{t_{p}+f_{p}+f_{n}+t_{n}}$$

(4) $$Specificity=\frac{t_{n}}{f_{p}+t_{n}}$$

where the terms $f_{p}$ “false-positive” and $f_{n}$"false-negative” stands in contrast to $t_{p}$ “true positive” and $t_{n}$ “true negative,” respectively.

**Fig. 4:**
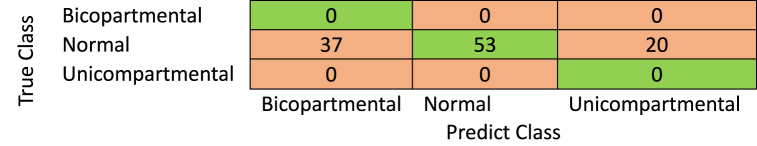
The proposed model is without data augmentation.

**Fig. 5:**
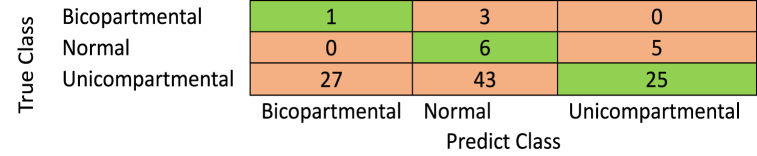
Alexnet with data augmentation.

The confusion matrix of the proposed method is shown in Fig. 4, while Table 1 summarizes the obtained measures (see Fig. 5).

**Table 1:** Performance measure without augmentation.

	**Accuracy**	**specificity**
**The proposed model**	65	66.67

According to the results obtained and summarized in Table 1, The accuracy of the proposed method was 65 %, while the specificity was 66.67 without augmentation. The proposed method constructs the deep learning model based on the dataset images. Instead of constructing a stacked layer deep learning model, the proposed method refined the residual learning to overcome the problem of a lack of images in the dataset. The proposed method fails to detect bicompartmental.

### 3.2 Discussions

The previous results show that the performance measures of the proposed method still need some enhancement. Because the proposed method fails to detect and classify bicompartmental, we suggest augmenting the dataset images and comparing the obtained results with others.

To our knowledge, no recently published paper on the same dataset exists. Therefore, we used a pre-trained model with transfer learning to compare the obtained measures using the proposed deep learning model against the pre-trained model. Here, Alexnet, shuffelnet, mobilenetv2, darknet53, and googlenet were used with transfer learning to compare the proposed method against others. The last two layers have been replaced with new, fully connected classification layers. In addition, all the previous layers were frozen to keep all the weights of these layers fixed without any change. Finally, the last two layers have been trained using the knee images (see Fig. 9).

**Fig. 6:**
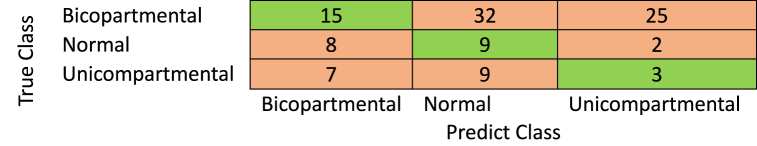
Darknet53 with data augmentation.

**Fig. 7:**
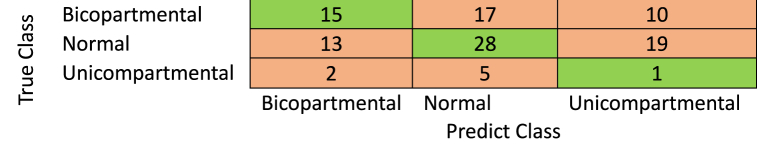
Mobilenetv2 with data augmentation.

**Fig. 8:**
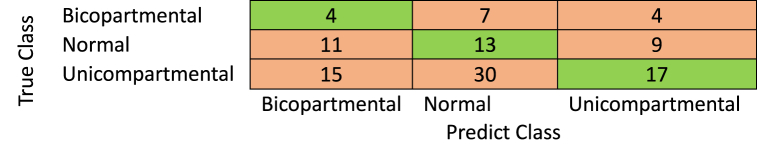
Shuffelnet with data augmentation.

**Fig. 9:**
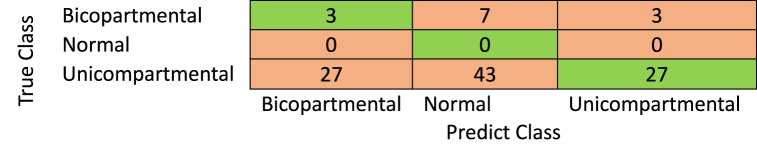
Googlenet with augmentation.

**Fig. 10:**
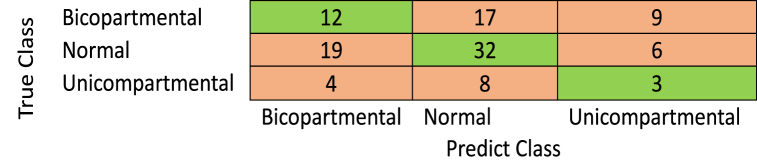
The proposed model with data augmentation.

**Fig. 11:**
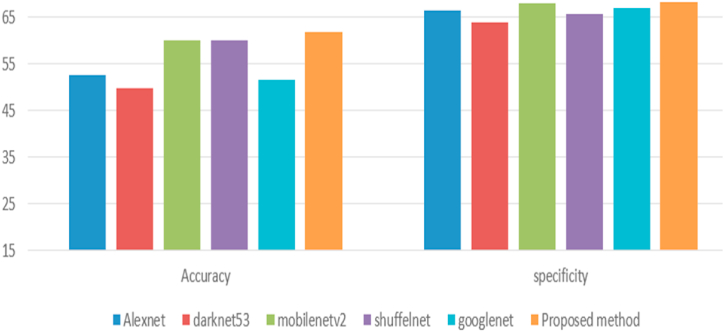
Clarification of the obtained results for the proposed method and Alexnet with transfer learning after data augmentation.

Before training the proposed model, some limitations and challenges must be added to relatively little data that can be accessed readily. So, we use various augmentation techniques, including random vertical and horizontal flips, random vertical and horizontal shifts, and random rotation angles between 0 and 360. There are requirements for augmentation, such as overcoming the limitation of the dataset images and generalizing the CNNs model to overcome the issue of overfitting and underfitting. The confusion matrices for modified pre-trained CNNs and the proposed method are shown in Fig. 5, Fig. 6, Fig. 7, Fig. 8, Fig. 9, Fig. 10. Table 2 summarizes the measures obtained, while Fig. 11 depicts the results.

**Table 2:** Performance measure with data augmentation.

	Accuracy	specificity
Alexnet	52.72	66.33
Darknet53	49.7	64
Mobilenetv2	60	68
Shuffelnet	59.94	65.67
Googlenet	51.52	67
**The proposed model**	**61.81**	**68.33**

The previous results show that the proposed method's performance measures are better than all pre-trained CNNs with transfer learning in detecting the three classes, bicompartmental, normal, and unicompartmental, as shown in Fig. 4. Although the proposed model was not pre-trained, it performed better than pre-trained CNNs. However, these measures are still unacceptable for many reasons. Based on radiographic findings, we will require baseline diagnostic imaging and follow-up clinical evaluations to identify uni-bicompartmental knee osteoarthritis. These assessments can be costly as well as challenging to obtain. These issues lead to limited data. Even after augmentation, the features are still the same. Secondly, there were no precomputed features, segmentations of the X-ray images, or processed images.

The limitation of this study is that the dataset contains a few knee images and the extraction of some redundant information, which degrades classification accuracy and lengthens computation, are the main limitations of this study. In the future, the dataset of knee images must increase. In addition, Bayesian optimization can be used to solve this problem.

## 4 Conclusions

This study proposes a deep redefined residual network architecture to classify knee OA as uni-bicompartmental. The model utilizes convolutional, pooling, and batch normalization layers to extract features from knee X-ray images. We used a dataset containing 733 knee X-ray images (331 normal Knee images, 205 unicompartmental, and 197 bicompartmental knee images). This study shows satisfactory results with 61.81 % and 68.33 % for accuracy and specificity, respectively. Then, we compare the performance of the proposed model with pre-trained CNN models (Alexnet, shufflenet, mobilenetv2, darknet53, and googlenet) using transfer learning. Results show that the proposed model outperforms the pre-trained models regarding accuracy and specificity. The proposed study could help orthopedic surgeons and related healthcare specialists interpret knee OA, especially in remote areas with insufficient qualified physicians. It can also assist in classifying the pathology into uni-bicompartmental, thus facilitating its treatment with either unicompartmental arthroplasty or bicompartmental (total) knee replacement.

## Data availability

Data is available on request from the authors.

## CRediT authorship contribution statement

**Soaad M. Naguib:** Writing – original draft, Validation, Formal analysis, Data curation, Conceptualization. **Mohamed A. Kassem:** Writing – original draft, Visualization, Software, Methodology, Conceptualization. **Hanaa M. Hamza:** Visualization, Resources, Project administration, Data curation. **Mostafa M. Fouda:** Resources, Project administration, Funding acquisition. **Mohamed K. Saleh:** Visualization, Validation, Data curation, Conceptualization. **Khalid M. Hosny:** Writing – review & editing, Supervision, Methodology, Conceptualization.

## Declaration of competing interest

The authors declare that they have no known competing financial interests or personal relationships that could have appeared to influence the work reported in this paper.
